# Supplementary material for: Surface plasmon resonance spectroscopy of single bowtie nano-antennas using a differential reflectivity method
Source: Sci Rep. 2016 Mar 23;6:23203. doi: 10.1038/srep23203 (PMC4804333; doi:10.1038/srep23203)
Supplement: Supplementary Information [file srep23203-s1.pdf]

Surface plasmon resonance spectroscopy of single bowtie nano-antennas using a differential reflectivity method

- Supplementary Material -

M. Kaniber[1,*], K. Schraml[1], A. Regler[1], J. Bartl[1], G. Glashagen[1], J. Wierzbowski[1] & J. J. Finley[1,2]

[1] Walter Schottky Institut and Physik Department, Technische Universität München, Am Coulombwall 4, 85748 Garching b. München, Germany

[2] Nanosystems Initiative Munich (NIM), Schellingstraße 4, 80799 München, Germany.

* Correspondence and requests for materials should be addressed to M.K. (kaniber@wsi.tum.de)

KEYWORDS: PLASMONICS, BOWTIE, OPTICAL ANTENNAS, SEMICONDUCTOR, REFLECTIVITY SPECTROSCOPY, FIELD ENHANCEMENT

In Figure SM 1(a), we present a selection of scanning electron microscopy images of lithographically defined Au bowtie nanoantennas on a semiconducting GaAs substrate. The top (bottom) row shows bowtie nanoantennas for constant nominal triangle size $s_0 = 110\mathrm{nm}$ (gap size $g_0 = 10\mathrm{nm}$) and increasing g_0 (s_0) between $5\mathrm{nm}$ ($90\mathrm{nm}$) and $50\mathrm{nm}$ ($150\mathrm{nm}$) from left to right, respectively. The highly reproducible fabrication process is further supported by the histograms plotted in Figure SM 1(b), representing the number of individual bowtie nanoantennas as a function of triangle size deviation $\Delta_s \equiv s - s_0$ and gap size deviation $\Delta_g \equiv g - g_0$ in the left and right panel, respectively. Both histograms for Δ_s and Δ_g are well described by a Gaussian distribution $y(x) = \frac{A}{\sigma\sqrt{2\pi}}\exp(-\frac{(x-\mu)^2}{\sigma^2})$ where μ and σ denote the expectation value and the standard deviation, respectively. From the fits of the triangle size and gap size histograms, we obtain narrow distributions indicated by the small values of the corresponding $\sigma_s^{GaAs} = 2.6\mathrm{nm}$ and $\sigma_g^{GaAs} = 3.8\mathrm{nm}$. This means in particular that $\sim 95.4\%$ of the fabricated triangles exhibit deviations in triangles size and gap size of less than $2\sigma_s^{GaAs} = 5.2\mathrm{nm}$ and $2\sigma_g^{GaAs} = 7.6\mathrm{nm}$ from the nominal values, respectively.

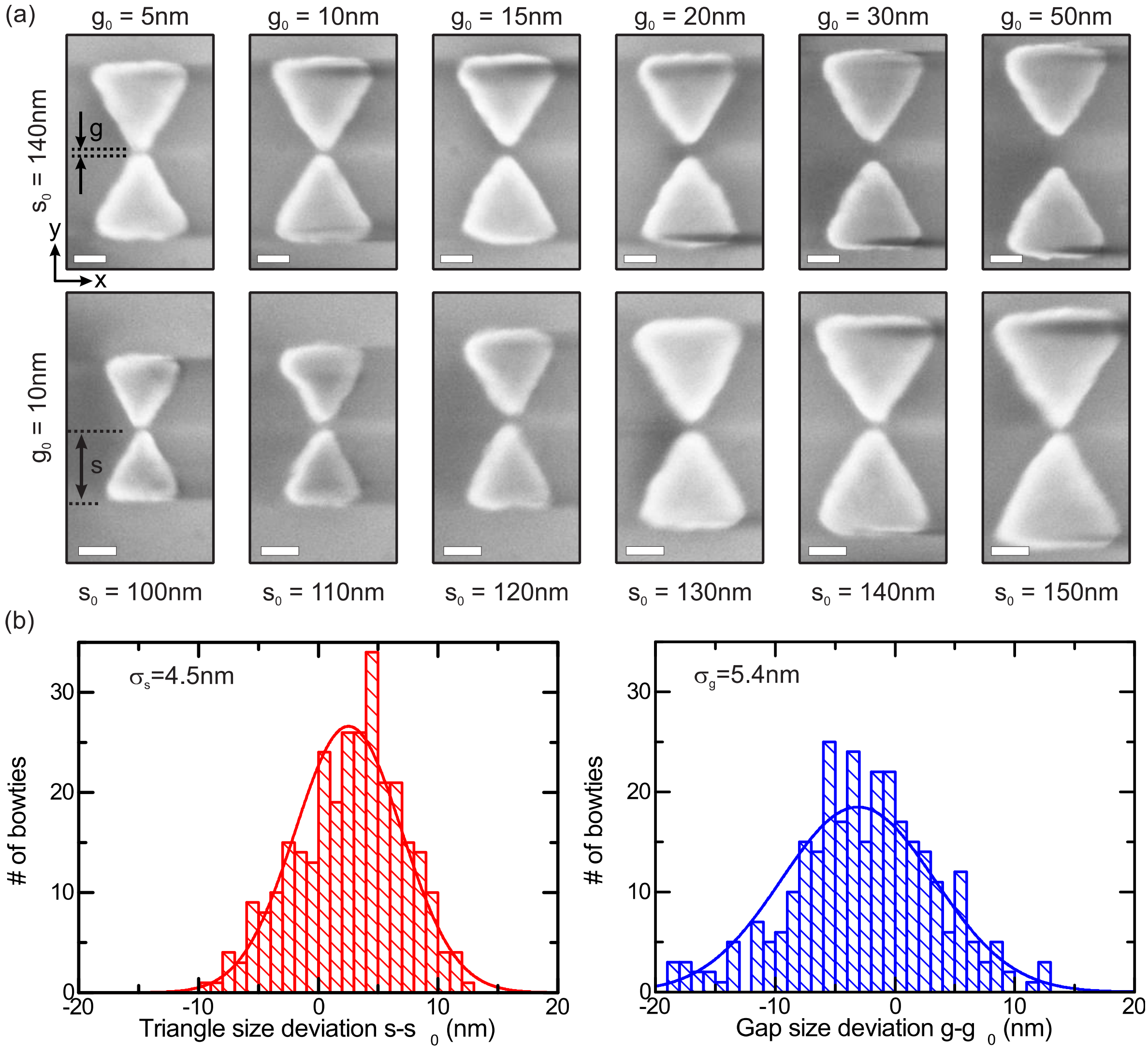

Figure SM 1 (a) Top row: Scanning electron microscopy images of individual bowtie nanoantennas on a GaAs substrate for $s_0 = 110nm$ as a function of nominal gap size $5nm < g_0 < 50nm$ from left to right, respectively. Bottom row: Scanning electron microscopy images of individual bowtie nanoantennas on a GaAs substrate for $g_0 = 10nm$ as a function of nominal triangle size $90nm < s_0 < 150nm$ from left to right, respectively. Scale bar, $50nm$. **(b)** Left panel: Statistical analysis of the number of bowtie nanoantennas as a function of the triangle size deviation $s - s_0$. Right panel: Statistical analysis of the number of bowtie nanoantennas as a function of the gap size deviation $g - g_0$.

In Figure SM 2 (a) and (b), we present the measured differential reflectivity spectra $\Delta R/R_{off}$ and the corresponding simulated scattering cross-section σ as a function energy for bowtie nanoantennas with gap size $6nm < g < 29nm$ and triangle size $s_0 = 140nm$ on a glass substrate. In Figure SM 2 (c) and (e), we present the measured differential reflectivity spectra $\Delta R/R_{off}$ as a function energy for bowtie nanoantennas with triangle size $100nm < g_0 < 150nm$ and gap size $g_0 = 10nm$ and bowtie nanoantennas with gap size $10nm < g < 37nm$

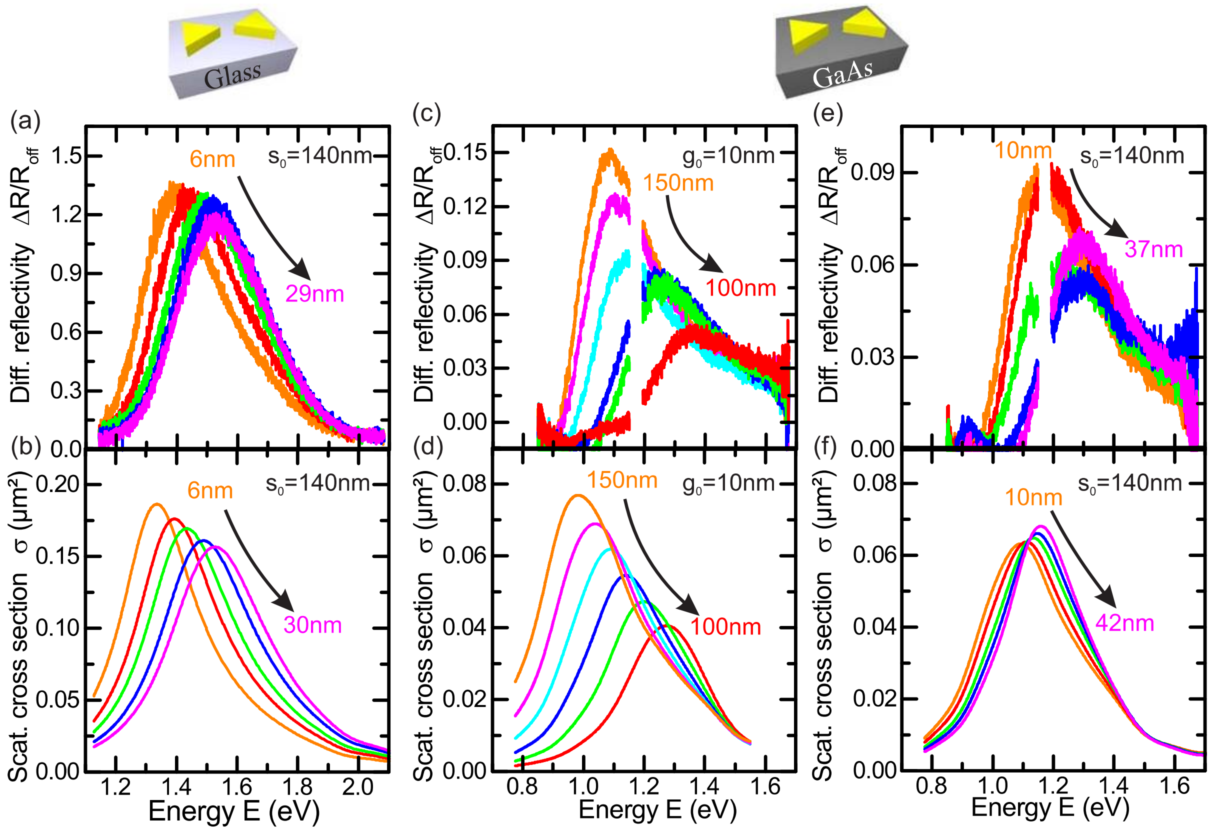

Figure SM 2 (a) Differential reflectivity $\Delta R/R_{off}$ and **(b)** numerically simulated scattering cross-section σ as a function of energy E for gap sizes $6nm < g < 29nm$ and $s_0 = 140nm$ on a glass substrate. **(c)** Differential reflectivity $\Delta R/R_{off}$ and **(d)** numerically simulated scattering cross-section σ as a function of energy E for triangle sizes $100nm < s_0 < 150nm$ and $g_0 = 10nm$ on a GaAs substrate. **(e)** Differential reflectivity $\Delta R/R_{off}$ and **(f)** numerically simulated scattering cross-section σ as a function of energy E for gap sizes $10nm < g < 37nm$ and $s_0 = 140nm$ on a GaAs substrate.

and triangle size $s_0 = 140nm$ on a GaAs substrate, respectively. The corresponding simulated scattering cross-sections σ are presented in panel (d) and (f) of Figure SM 2.

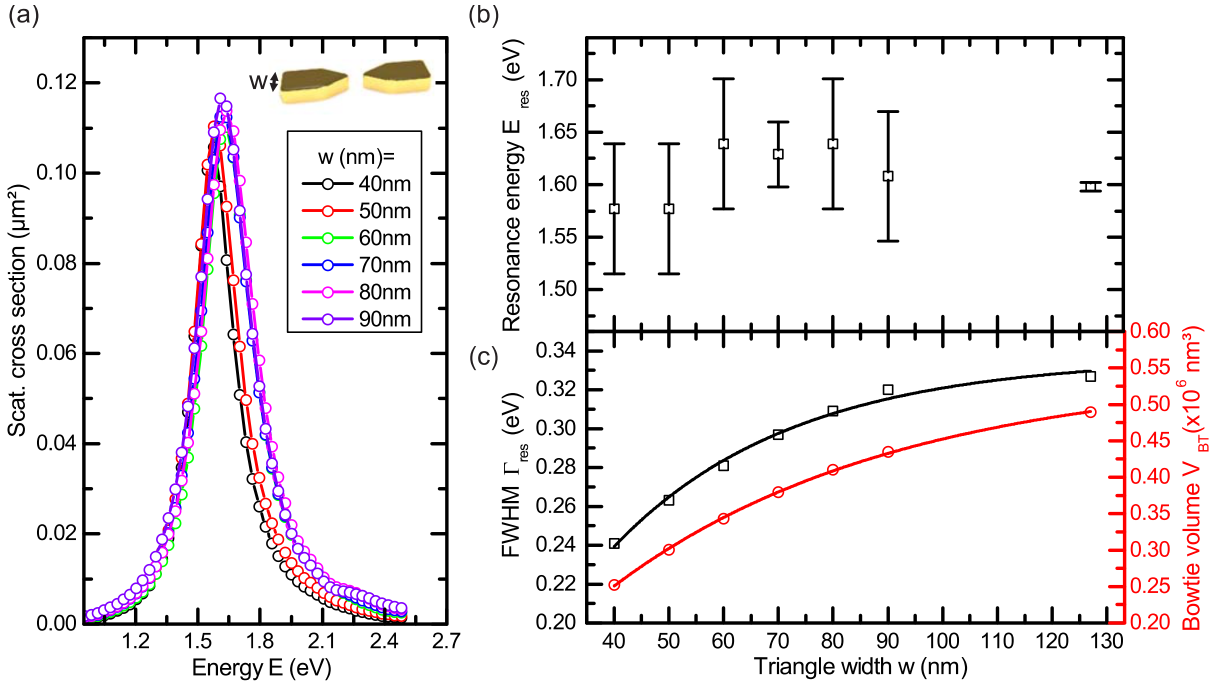

Figure SM 3 (a) Numerically simulated scattering cross-section σ as a function of energy E for truncated bowtie nanoantennas with triangle size $s_0 = 110nm$, gap size $g_0 = 10nm$ and triangle width $40nm < w < 90nm$. **(b)** Localised surface plasmon polariton resonance E_{res} as a function of triangle width w. **(c)** Full width at half maximum Δ_{FWHM} (black) and truncated bowtie nanoantenna volume V as a function of triangle width w.

In Figure SM 3 (a), we present numerical simulations of the scattering cross-section σ as a function of energy E for a truncated bowtie nanoantenna. Here, we define the triangle width w as the based length of one nanotriangle as schematically depicted in the inset of Figure SM 3 (a). We observe experimentally that the localised surface plasmon polariton resonance energy E_{res} stays almost unaffected at an average value of $\langle E_{res} \rangle = 1.601eV \pm 0.027eV$ when decreasing the triangle width from $w = 127nm$ (pristine equilateral triangle size $s_0 = 110nm$) to $w = 40nm$, as shown in Figure SM 3 (b). This is due to the fact that the resonance energy of such dimer-type nanoantennas is predominantly governed by the total length of the nanoantenna (i.e. $2 \cdot s_0 + g_0$) and the additional lowering in energy due to the pronounced near-field coupling. In strong contrast, our simulations show that the full width at half maximum Γ_{res} decreases by a factor $\sim 1.3\times$, when decreasing the triangle width from $w = 127nm$ (pristine equilateral triangle) to $w = 40\mathrm{nm}$, as shown by the black data set in Figure SM 3 (c). This decrease in $\Delta\Gamma$ gives rise to a decrease in losses due to the reduction of the amount of metal as shown by the red data set in Figure SM 3 (c). Our results are in good qualitative agreement with simulations of 'two-wire gap'-like antennas as presented in Ref. [9].
